# Renal Cell Carcinoma Metastatic to Thyroid Gland, Presenting Like Anaplastic Carcinoma of Thyroid

**DOI:** 10.1155/2013/651081

**Published:** 2013-04-10

**Authors:** Khalid Riaz, Mutahir A. Tunio, Mushabbab AlAsiri, Asim Ali Elbagir Mohammad, Muhammad Mohsin Fareed

**Affiliations:** ^1^Radiation Oncology, Comprehensive Cancer Center, King Fahad Medical City, Riyadh 59046, Saudi Arabia; ^2^Histopathology and Cytopathology, King Fahad Medical City, Riyadh 59046, Saudi Arabia

## Abstract

*Background.* Renal cell carcinoma (RCC) has unpredictable and diverse behavior. The classic triad of hematuria, loin pain, and abdominal mass is uncommon. At time of diagnosis, 25%–30% of patients are found to have metastases. Bones, lungs, liver, and brain are the frequent sites of metastases. RCC with metastasis to the head and neck region and thyroid gland is the rarest manifestation and anaplastic carcinoma behaving metastatic thyroid mass is an extremely rare presentation of RCC. *Case Presentation.* A 56-year-old Saudi man with past history of right radical nephrectomy 5 years back presented with 3 months history of rapid increasing neck mass with dysphagia, presenting like anaplastic thyroid carcinoma. Tru-cut biopsy turned out to be metastatic renal cell carcinoma. Patient was treated with radiation therapy 30 Gy in 10 fractions to mass. Patient died 4 months after the discovery of anaplastic thyroid looking metastasis. *Conclusion.* Rapidly progressing thyroid metastases secondary to RCC are rare and found often unresectable which are not amenable to surgery. Palliative radiotherapy can be considered for such patients.

## 1. Introduction

Renal cell carcinoma (RCC) has unpredictable and diverse behavior. Due to widespread use of modern imaging, the incidence of RCC over last 20 years has progressively increased [1]. About 30%–50% of patients are found to have metastases at the time of diagnosis. While bone, lymph nodes, lungs, and brain constitute expected metastatic sites, metastasis may turn up at the unusual locations (skin, testis, maxillary antrum, and cervix) [2–4]. 

Metastasis of RCC to the head and neck region is rare manifestation [5, 6]. The breast and lung cancers are known to metastasize to the thyroid and in some cases they are detected only at autopsy [6]. 

The common presenting symptoms of metastatic RCC to the thyroid gland are enlarged solitary or multiple neck swellings, dysphagia, and dyspnea depending on the location and the extent of invasion by metastatic deposits [7]. To differentiate a primary thyroid malignancy from RCC, the clinical and radiographic findings are often nonspecific; however, the histopathological and immunohistochemical features are sufficiently distinctive.

Herein, a rare case of thyroid gland metastasis presenting like anaplastic thyroid cancer following an open right radical nephrectomy five years back in a 56-year-old male with a stage pT2N0 M0 and Fuhrman grade II RCC is reported.

## 2. Case Presentation

**Figure 1:**
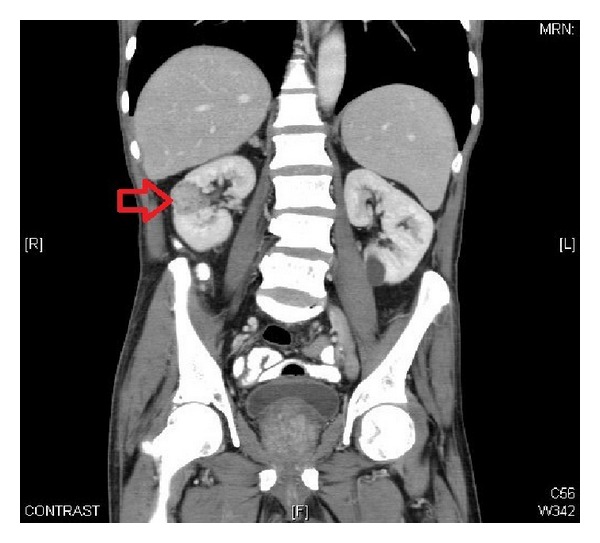
Computed tomography of abdomen (coronal view) showing right kidney mid pole mass turned out as renal cell carcinoma upon radical nephrectomy.

A 56-year-old Saudi man presented during routine followup with neck swelling and hoarse voice. He had noticed this swelling for 3 months and it had been rapidly increasing in size over a week causing dyspnea and dysphagia to solids. His previous medical history revealed that, five years back, he underwent left radical nephrectomy; the histopathological findings were consistent with papillary cell type renal cell carcinoma (Figure 1). Tumor was not infiltrating through the capsule. Hilar vessels, margins, and bladder cuff were free of tumor. Stage was made pT2N0 M0. He had no other medical comorbidities and no history of smoking and her weight was stable. 

**Figure 2:**
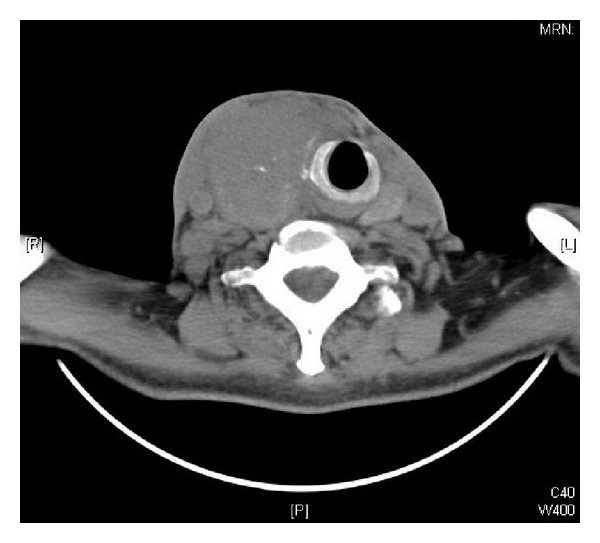
Computed tomography of neck showing left thyroid mass of size 8 × 7.5 cm.

On physical examination, there was hard fixed palpable in thyroid gland. The rest of examination was normal. Computed tomography (CT) scan of his neck was performed which revealed a mass of 8 × 7.5 cm size in right lobe of thyroid gland with vascular enhancement (Figure 2). No cervical lymphadenopathy was found and postnephrectomy renal bed was clear. The bone scan and CT chest revealed no other distant metastases. Differential diagnosis was anaplastic thyroid carcinoma or lymphoma. 

**Figure 3:**
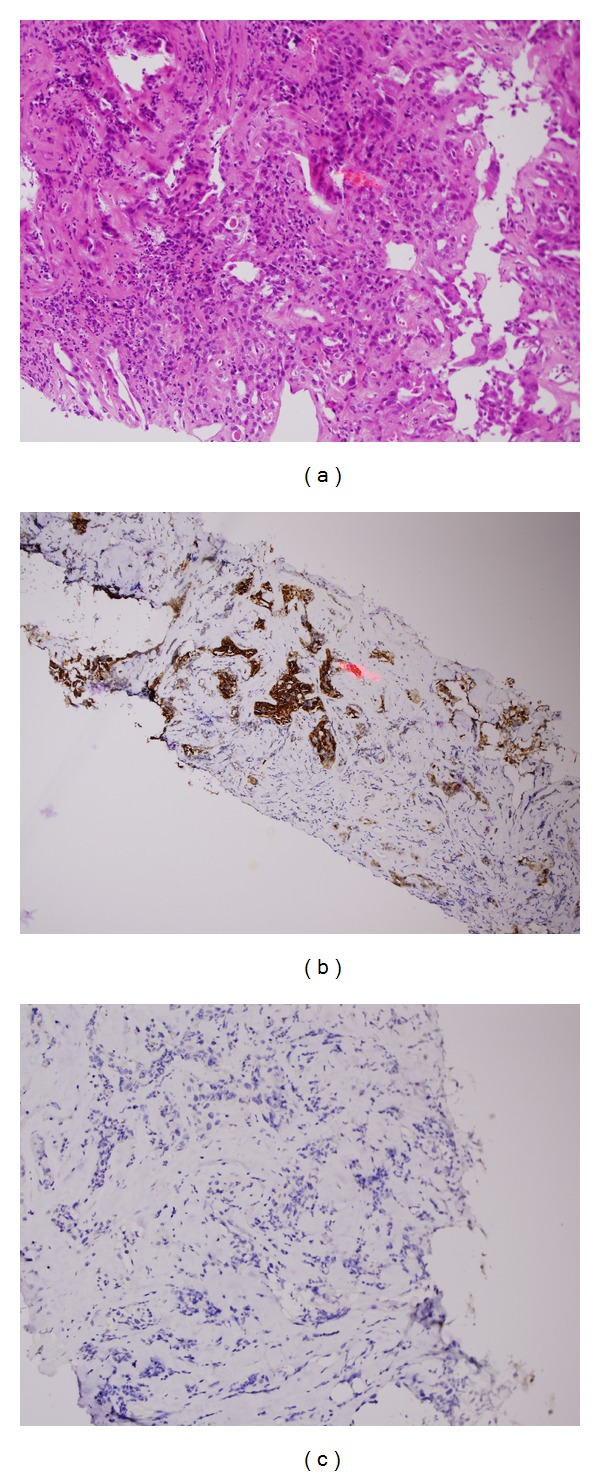
(a) Hematoxylin and Eosin staining of tru-cut biopsy of thyroid mass showing clear cell histology (b) positivity for CD10 and (c) negativity for TTF-1, consistent with metastatic renal carcinoma.

Subsequently tru-cut biopsy was taken from thyroid mass, which revealed clear cell histology, and immunohistochemistry revealed CD10 positivity and TTF-1 negativity (Figure 3). Findings were consistent with metastatic RCC. Patient was given palliative radiotherapy 30 Gy in 10 fractions to relieve the obstructive symptoms as tumor was unresectable and then he was started on sunitinib 50 mg orally daily. Patient died 4 months after the discovery of thyroid metastasis. 

## 3. Discussion

Metastasis to the thyroid gland is an uncommon manifestation. However, autopsy results show that 1.9% to 22.4% of patients with generalized malignancies have metastasis to the thyroid gland [7]. According to one large autopsy series, malignant melanoma (39%) and breast carcinoma (21%) and lung account for the largest number of tumors metastasizing to the thyroid gland as part of widely disseminated disease (excluding lymphoma and leukemia) and thyroid gland metastases from renal cell carcinoma are rarest [8] and further anaplastic like presentation of thyroid metastasis is extremely rare. 

Although micrometastases are present in about 25% of RCCs at the time of the diagnosis of the primary malignancy (synchronous), metastatic disease can develop as part of the latency of the tumor with delayed development of metastases after many years of dormancy (metachronous) as seen in our patient [9]. 

The thyroid gland is one of the most vascularized organs in the body and one would expect it to be the site of metastatic disease. It has been suggested that the thyroid gland, when altered by goiter, neoplasms, or thyroiditis, is more vulnerable to metastatic growth due to metabolic changes with a decrease in oxygen and iodine content [10]. 

Surgical treatment of patients with solitary thyroid gland metastases is recommended because of the unusually good prognosis in patients reported in the literature when they were treated with definitive surgical therapy with mean 5-year survival rate 30–60% [11]. However our patient was treated with radiation therapy as the tumor was unresectable.

In conclusion, thyroid metastases secondary to RCC are rare and the importance of this case lies in the fact that, on initial presentation, it mimicked the rapid growth of anaplastic thyroid carcinoma, for such cases radiation therapy can be an option.
